# Machine Learning Models for Enhanced Estimation of Soil Moisture Using Wideband Radar Sensor

**DOI:** 10.3390/s22155810

**Published:** 2022-08-03

**Authors:** Akileshwaran Uthayakumar, Manoj Prabhakar Mohan, Eng Huat Khoo, Joe Jimeno, Mohammed Yakoob Siyal, Muhammad Faeyz Karim

**Affiliations:** 1 School of Electrical and Electronic Engineering, Nanyang Technological University (NTU), Singapore 639798, Singapore; akileshw001@e.ntu.edu.sg (A.U.); manoj.mohan@ntu.edu.sg (M.P.M.); eyakoob@ntu.edu.sg (M.Y.S.); 2 Institute of High Performance Computing (IHPC), A*STAR, Singapore 138632, Singapore; khooeh@ihpc.a-star.edu.sg; 3 NCS Pte Ltd., 5 Ang Mo Kio Street 62, NCS Hub, Singapore 569141, Singapore; joe.jimeno@ncs.com.sg

**Keywords:** KNN, linear regression, microwave radar, neural network, SVM, soil moisture, volumetric water content

## Abstract

In this paper, machine learning models for an effective estimation of soil moisture content using a microwave short-range and wideband radar sensor are proposed. The soil moisture is measured as the volumetric water content using a short-range off-the-shelf radar sensor operating at 3–10 GHz. The radar captures the reflected signals that are post processed to determine the soil moisture which is mapped to the input features extracted from the reflected signals for the training of the machine learning models. In addition, the results are compared and analyzed with a contact-based Vernier soil sensor. Different machine learning models trained using neural network, support vector machine, linear regression and k-nearest neighbor are evaluated and presented in this work. The efficiency of the model is computed using root mean square error, co-efficient of determination and mean absolute error. The RMSE and MAE values of KNN, SVM and Linear Regression are 11.51 and 9.27, 15.20 and 12.74, 3.94 and 3.54, respectively. It is observed that the neural network gives the best results with an R2 value of 0.9894. This research work has been carried out with an intention to develop cost-effective solutions for common users such as agriculturists to monitor the soil moisture conditions with improved accuracy.

## 1. Introduction

The estimation of soil moisture has gained significant interest amongst researchers recently. The different applications where moisture detection is crucial are hydro-meteorological, agricultural research and climate change [1]. It is essential to detect the soil moisture as it replenishes the water table, controls the penetration of the quantity of water into the soil and is a significant contributor to channel flow and surface runoff [2]. To detect the soil moisture, contact sensors and remote sensing sensors are widely used [3]. Contact sensors require the manual placement of the sensor into the soil. This process can be tedious, as both setting up and maintaining the soil moisture sensor is labor intensive, especially for vast areas.

Remote sensing eliminates the disadvantage of the previous method and is the process in which the physical characteristics of an area are detected and monitored from a certain distance above the ground. Microwaves are electromagnetic radiations with frequencies in the range of 300 MHz–300 GHz and are commonly used in remote sensing due to its long wavelength and easy penetration capabilities through different materials. The infiltration reduces with the rise in the amount of moisture content in the soil and vegetation. Additionally, soil moisture can be determined at any time and in almost all weather and environmental conditions [4,5,6].

Microwave remote sensing can be classified into active and passive microwave remote sensing [6]. First, active microwave remote sensing uses radars to emit microwaves to determine the soil moisture. The microwaves are either absorbed or reflected from the surface of the soil. The characteristic of the soil is then determined by comparing the emitted rays from the radar and the reflected rays from the soil [7,8]. Second, passive microwave sensing does not use a radar to emit microwaves but captures the microwave radiations from the ground surface [9].

In the remote sensing community, the machine learning methods for the estimation of earth system parameters has recently received much interest [10]. Machine learning techniques are quick and simple to apply and are completely data driven. These techniques usually are aimed at creating a general function based on the internal parameters and map the data variable with the desired target variable. Machine learning was used to predict the soil moisture with the data collected from the satellite-based Synthetic Aperture Radar (SAR) [11,12,13]. However, the soil moisture values from the ground data and the satellite values are very different, as mentioned in [3]. Support vector regression technique was investigated to estimate the soil moisture content effectively [14,15]. Soil moisture was determined using ultra-wideband sensor data with the help of fuzzy logic systems [3]. This algorithm was used to track the trend of time series data to compute the moisture content. The above-mentioned research works have been performed on data available from radar/weather satellites. Several machine learning models were analyzed in the prediction of soil moisture along the Red River Valley in North Dakota and Minnesota, US. The data sets collected were mainly from the local weather stations and the agricultural lands [16]. Twenty microstations were used to collect the data in the Translyvanian depression in Romania for the measurement of soil moisture [17] and several machine learning algorithms were compared. These papers collected the data and measured the soil moisture over the large areas.

However, using machine learning algorithms on data collected with a short-range radar opens the possibility of new and effective applications with accurate results, and this has been explored in this research work. The short-range radars can be easily mounted onto vehicles, agricultural machinery, and drones. The soil moisture can be determined for a small piece of land rather than over a large area. Even though there are few methods [18] where cameras were used to measure the soil moisture, but their performance during night is not known.

In this paper, accurate soil moisture is determined using different machine learning models with a short-range and wideband radar sensor operating in a range of 3–10 GHz. The efficiency of the models is computed using root mean square error, co-efficient of determination and mean absolute error. The paper is organized into three different sections. Section 2 provides details of the radar sensor used for the detection of moisture and the method used to collect data for training the machine learning algorithm. It also explains in detail how the experiments were performed in the collection and training of the data. Section 3 provides the detailed discussion of the results and the performance of the proposed solution.

## 2. Materials and Methods

This section introduces the system setup and methods for measuring the soil moisture using the wideband microwave radar.

### 2.1. System Setup

**Figure 1:**
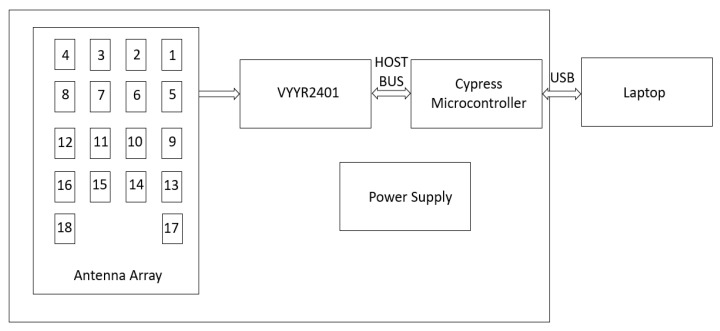
Block diagram of Walabot radar sensor.

We have selected an off-the-shelf Walabot radar sensor that operates at 3.3–10.3 GHz frequency [19]. This radar has been previously used to determine dielectric constant in concrete and drywall environments using short-range imaging [19], and we also adopted the same principle to determine the dielectric constant of the soil. The wavelength in these frequency ranges (3.3 GHz–10.3 GHz) is from 0.09 m to 0.029 m, which is bigger than the size of the soil particles. Due to this, the soil appears as an effective uniform layer to the waves from the radar. Also, the wide bandwidth of the radar helps to get the variation of the dielectric constant over a large frequency range enabling the accurate measurement of the soil moisture. The radar contains a 2D antenna array consisting of 18 antennas as shown in Figure 1. It works by transmitting the microwave signal from the selected antennas and records the signals reflected from the surface of the object. Apart from Walabot radar, some of the other materials used are the Styrofoam plate to hold the soil sample, an aluminum metal for reference, a tripod stand, PC, and absorbers to eliminate unwanted reflected radiation. Also, a Vernier soil moisture sensor with a LabQuest 2 interface is used for the experiment [20]. The power level of the sensor decides the distance between the target and the radar.

### 2.2. Methods and Approach

The soil moisture is the amount of water present in the soil and can be represented as Volumetric Water Content (VWC), $\theta_{v}$, which is the ratio of the volume of water to the total volume of the soil [21] as given by Equation (1),

(1) $$\theta_{v}=\frac{\mathrm{Volume}\;\mathrm{of}\;\mathrm{water}}{\mathrm{Total}\;\mathrm{volume}}$$

The maximum value of volumetric water content in a soil can be 45%, beyond which there are no more air pockets to hold moisture and this soil is referred to as saturated [17]. Additionally, it also provides the method used to determine the data for training the machine learning model.

A two-step approach is adopted to determine the volumetric water content. First, using dielectric constant, and then using a regression-based model. In the first process, $\theta_{v}$ is determined by measuring the dielectric constant or relative permittivity $\varepsilon_{r}^{\prime }$ of the soil. Dielectric constant is the capability of the material to store electric energy. It has constant value that changes with change in frequency, moisture or properties of materials like density and temperature [21]. Thus, there is a change in dielectric constant of the soil with a change in moisture content. The dielectric constant can be determined by Equation (2):

(2) $$\varepsilon_{r}^{\prime }={\left(\frac{1+\left|F\left(\mathrm{sample}\left(t\right)-\mathrm{ref}\left(t\right).w\left(t\right)\right)/F\left(\mathrm{metalref}\left(t\right)-\mathrm{ref}\left(t\right).w\left(t\right)\right)\right|}{1-\left|F\left(\mathrm{sample}-\mathrm{ref}\left(t\right).w\left(t\right)\right)/F\left(\mathrm{metalref}\left(t\right)-\mathrm{ref}\left(t\right).w\left(t\right)\right)\right|}\right)}^{2}$$

where, F represents Fourier transform, w(t) represents the windowing function and $\varepsilon_{r}^{\prime }$ is the dielectric constant of the soil.

**Figure 2:**
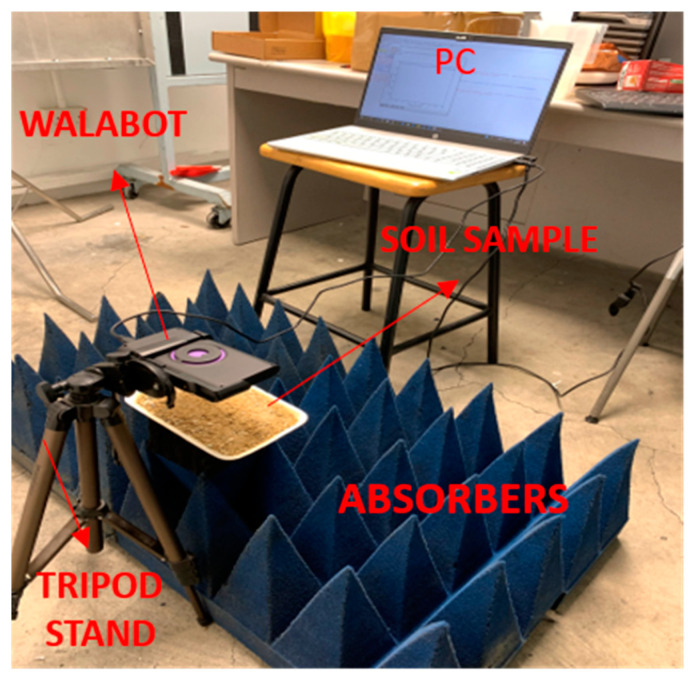
Experimental Setup.

The soil sample is taken from surface residual soil (grade VI) [22] found in our University’s (NTU) campus and it is of clayey silt type. This falls under the Jurong formation which is one of the four formations of the geology of Singapore and further details regarding the soil types in Singapore can be found in [22]. The percentage of fine particles for the residual soil in Jurong formation is 65%, with a minimum percentage of 38% and a maximum percentage up to 95% [23]. The particle diameter of the residual soil in Jurong formation varies from 1 × 10^−4^ mm to 70 mm (Figure 2 in [23]) with the particle diameters of sand, clay and silt at less than 10 mm; only gravel has a larger particle diameter. Therefore, the wavelength of the radar is still higher than the particle size, and the soil appears as an effective uniform layer to the waves. The soil sample is taken in the Styrofoam plate and the Walabot radar is placed at 10 cm above the sample. The distance of 10 cm is selected based on the sensitivity and the higher amplitude of the reflected waves detected by the radar. The Walabot radar is mounted on a tripod stand to capture the reflected signals of the sample and is connected via a USB port to the PC as shown in Figure 2. In this experiment, the antenna pair 5 and 12 has been chosen where 5 is the transmitter antenna and 12 is the receiver antenna. The programming is done in python with the help of Walabot API to determine the volumetric water content.

The short-range image profile is selected to quantify the dielectric constant of the soil sample. For the estimation of the dielectric constant of soil, no filter is required, and the arena settings are set based on the dimensions of the sample holder. The sample is placed above the microwave absorbers to minimize the reflection from the surroundings. The Walabot radar first captures the reflected signals of the metal reference which is an aluminum plate placed on top of the empty Styrofoam plate. The next step is to capture the reflected signals of the soil sample.

**Figure 3:**
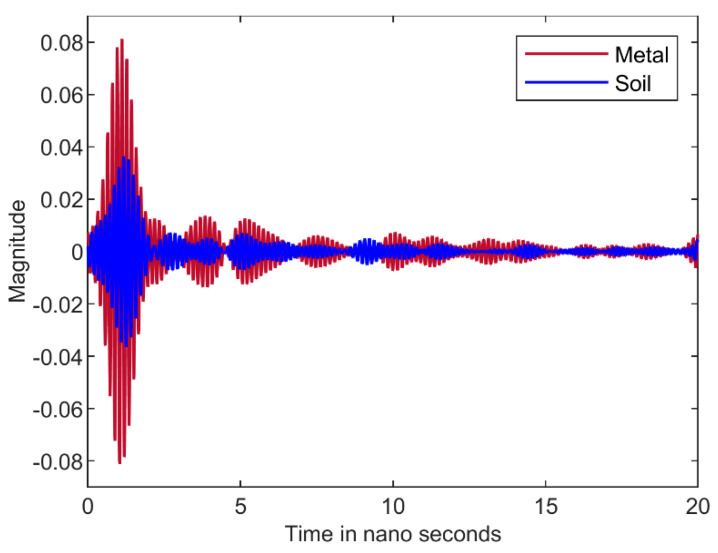
Reflected Signals of Metal and Soil.

Metal reflects almost all the signals that falls on it, so this serves as the reference and is then compared with the reflected signal from the soil as shown in Figure 3. Different windowing functions like Hanning, Hamming, Flat-top and Rectangular are applied [24] to extract the highest peak of the signals. Rectangular window generates better results in comparison with other windows and therefore is selected for the experiment. The geometric mean is calculated for the dielectric values [25] in the frequency range and this dielectric constant value is used to determine the volumetric water content.

The reflected signal values of the metal reference and the sample, as shown in Figure 3, are substituted in Equation (2) to determine the dielectric constant [25]. Finally, on determining the dielectric constant, the volumetric water constant can be computed using the Topps equation as given by Equation (3). This equation does not entail any prior information of the surface roughness or the soil texture [18]:

(3) $$\theta_{v}=-5{.3\;\times \;10}^{-2}+2{.92\;\times \;10}^{-2}\;\varepsilon_{r}^{\prime }-5{.5\;\times \;10}^{-4}\varepsilon_{r}^{\prime }{}^{2}+4{.3\;\times \;10}^{-6}\varepsilon_{r}^{\prime }{}^{3}$$

where, $\varepsilon_{r}^{\prime }$ is the dielectric constant of the soil and $\theta_{v}^{\;}$ is the volumetric water content.

In the second process, the volumetric water content values determined in the first process are input to the machine learning algorithms for training. Different machine learning algorithms like neural network, support vector machine, linear regression and k-nearest neighbor are implemented. The regression-based model fitting is used to find the relationship between the response variable against input variables. The response variable being volumetric moisture and the input variables being mean, standard deviation, mean of the instantaneous phase, skewness, mean absolute deviation, co-efficient of variance and kurtosis. Out of these input variables standard deviation, mean of the instantaneous phase, mean absolute deviation and kurtosis were chosen as input features. The data collected with the help of the experiment is used to train a model which is able to predict the soil moisture content accurately without any prior knowledge on the soil properties.

In this experiment, the VWC values are varied by gradually adding water to the soil sample to increase the VWC. The VWC of the soil is also measured using the Vernier soil moisture sensor and the values are displayed on the LabQuest2 interface. Few statistical parameters are extracted from the reflected signals. In this paper, the moisture values measured for the soil samples are −1.5% (dry soil), 2.2%, 19%, 25% and 37%, respectively. For every VWC value, 3 to 4 different readings are obtained and used for the purpose of training machine learning model.

## 3. Results and Discussion

**Figure 4:**
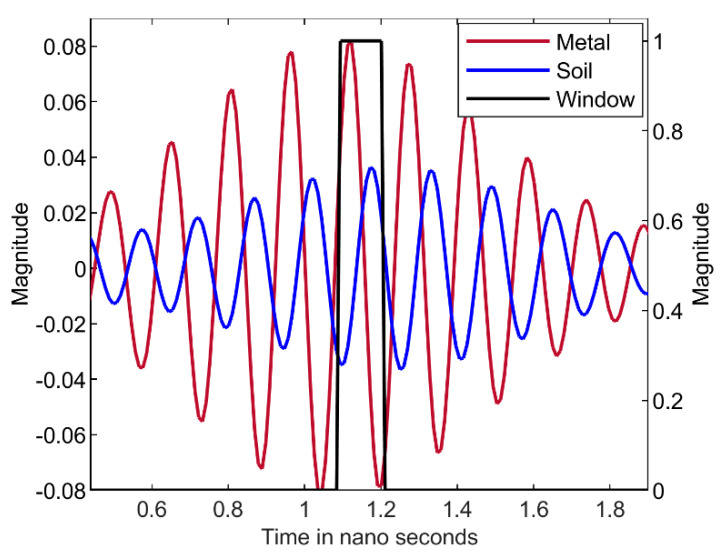
Rectangular Window applied for reflected signals of VWC of −1.5%.

**Figure 5:**
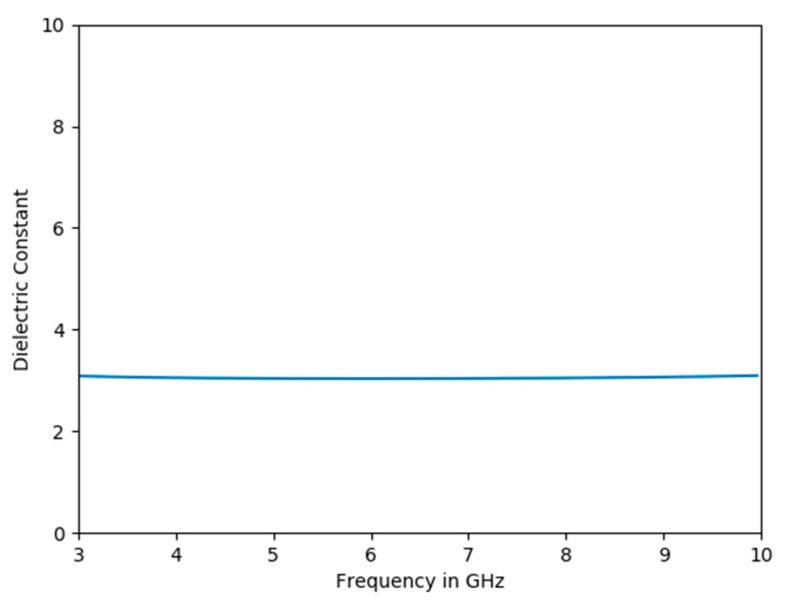
Dielectric Constant vs. Frequency for VWC of −1.5%.

The dielectric constant has been found using the procedure explained in Section 2. In Figure 4, the rectangular window is represented by a black color and the reflected signal from the metal and soil are in brown and blue colors (as in Figure 3). The reflected signals shown in Figure 3 are processed over the rectangular window for the soil with VWC −1.5%. The dielectric constant of the soil is determined and observed to be constant over the range of 3 GHz–10 GHz for the soil with VWC of −1.5% as shown in Figure 5. The result obtained after post processing gives the reflected signals and gives VWC values close to the values measured using the Vernier soil moisture sensor that is inserted into the soil as shown in Table 1. However, there is 3–5% of error for the values obtained from this experiment compared with the values from the Vernier soil moisture sensor.

To test the validity of this method, the same procedure above is repeated for the beach soil taken from the East Coast Park (ECP) of Singapore which falls under Kallang formation [22]. Table 2 shows the values of the soil moisture measured from using the Vernier sensor and also from the above method. The VWC values calculated from the microwave experiment are higher than the VWC values found out by the Vernier sensor for soil type in Table 1. But the VWC values from the microwave experiment are lower than the values found out by the Vernier sensor for the soil type in Table 2. This is due to the difference in texture and porosity between the two types. When proposing the Topp’s equation, [26] reported the different behavior of the dielectric constant between the clay soil and the sandy loam soil for the given VWC. Also, in [27], it is reported that for a given VWC, the value of permittivity increases if the soil porosity decreases. R- squared and root mean square error (RMSE) values of (3) were reported in [27] as 0.8189 and 0.078. The values from Table 2 are not used for the machine learning training since it is of the different soil type. The data suitable for machine learning from the experimentation results is considered as the data set (values from Table 1) which has 16 values. The 70–30% split validation is followed on the data set instead of 80–20% split so that there would be 30% of the data available for validation. The same 70–30% split was also used in [16].

**Figure 6:**
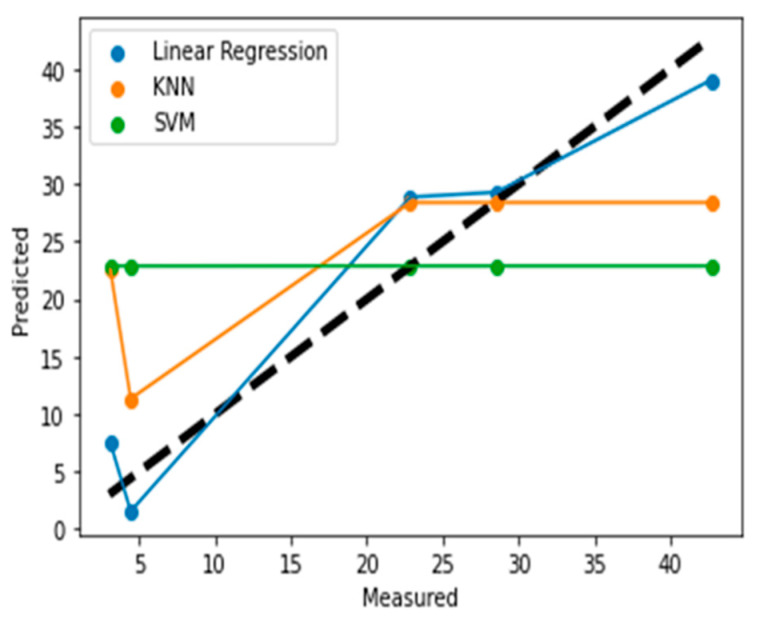
Measured vs. Prediction values for different machine learning algorithms with perfect fit represented as dotted black line.

The data set is used to train three different models, namely K Nearest Neighbor (KNN), Linear Regression and Support Vector Machine (SVM) in Python. Mean absolute error (MAE) and root mean square error (RMSE) are used to compare the VWC values that are predicted by the three models to demonstrate their efficiency. The measured vs. the predicted values of the three different models are shown in Figure 6. The results show that the linear regression model can predict better as compared to the KNN and SVM models.

**Figure 7:**
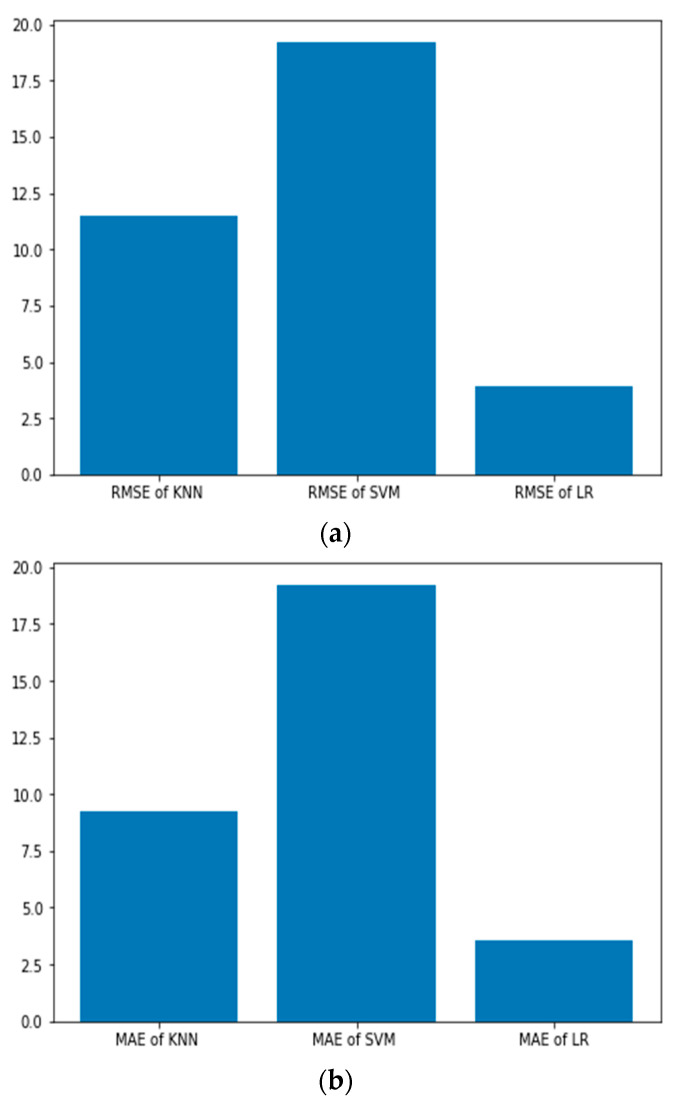

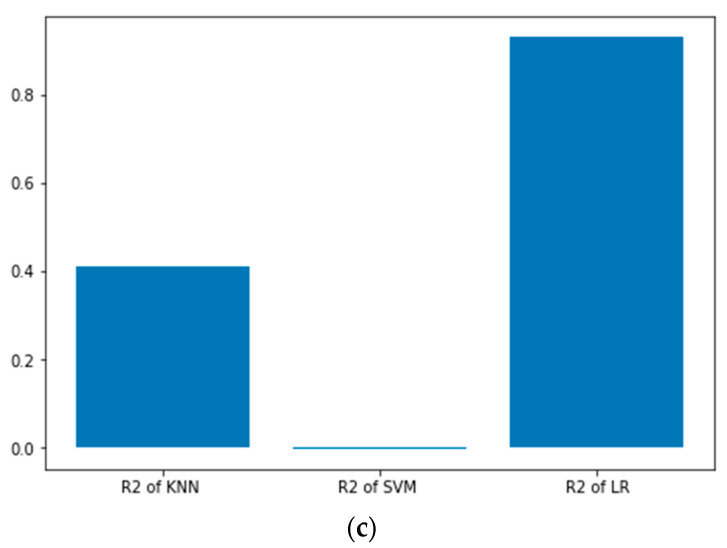
Performance comparison of three different models (**a**) RMSE (**b**) MAE (**c**) R^2^ values.

In Figure 7, the performance comparison of the three different models is depicted. The RMSE values of KNN, SVM and Linear Regression are 11.51, 15.20 and 3.94, respectively. The MAE values of KNN, SVM and Linear Regression are 9.27, 12.74 and 3.54, respectively. Furthermore, the R squared value, i.e., the coefficient of determination, a parameter that represents the goodness of fit and used in prediction, is determined for the different models [28,29]. The R^2^ value equal to 1 represents that the model perfectly fits the data.

**Figure 8:**
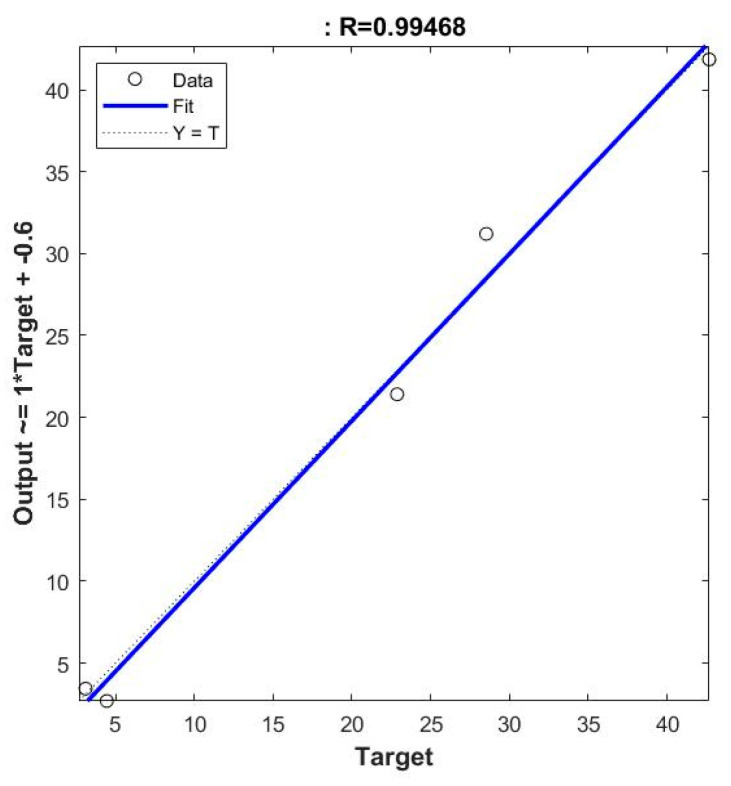
Neural network model. Measured vs. Predicted Values.

The R^2^ values obtained for KNN, SVM and Linear Regression models are 0.41, −0.02, and 0.93, respectively. Since the amplitude of the reflected signals decreases as the soil moisture increases, there is a linear relationship between the reflected signals and the soil moisture. KNN and SVM are the classification algorithms, so they work better if the number of target values is lesser (two or three) [30]. Here, the target range is VWC, whose value ranges from 3% to 40%. So, a regression algorithm (Linear regression in this case) works better [30]. Also, a neural network model was trained in the NN toolbox of MATLAB. The neural network type is Feed Forward Backprop and the training function is TRAINLM. The model is a two-layered network (Shallow Neural network) with 10 neurons in the first layer and with LEARNGDM as the adaption learning function and the performance function based on MSE. The measured vs. predicted values by the neural network model is shown using a regression plot in Figure 8. The R square value for neural network prediction is 0.9894 and it performs better than the other trained models. The performance metrics RMSE, MAE and R^2^ are chosen since the final output is a number and they are used for regression algorithms. These metrics were also used to compare the machine learning algorithms in [16] for soil moisture estimation but the accuracy alone was used in [17] to compare the algorithms for soil moisture prediction. The limitation of this study is that only one soil type is used, which is readily available and prevalent; however, later, this research will be extended to other conditions and soil types found in Singapore.

## 4. Conclusions

Machine learning models for an effective estimation of soil moisture content using a microwave short-range and wideband radar sensor have been presented. The soil moisture is measured as the volumetric water content using short-range off-the-shelf radar sensor operating at 3–10 GHz. The rectangular window has been applied to the reflected signals from both the metal and the soil. Thus, the dielectric constant of the soil has been found out for the frequency range of 3–10 GHz. The experiments have been carried out by varying the moisture content gradually to determine the dielectric constant and in turn, the volumetric water content to collect the data for the training of the models. The results from different models, namely KNN, SVM, linear regression and neural networks are analyzed with statistical parameters from the reflected signals serving as input features and volumetric water content as the output for the purpose of training. Comparison results show that linear regression is superior to the other two models with an R^2^ of 0.93. However, when a neural network model is trained using MATLAB, it outperforms the other models and achieves the best results with an R^2^ value of 0.9894, while predicting the soil moisture values as very close to the actual values. This research work can further be expanded in the future by exploring different windowing techniques, different soil types and by training the model with different features which could potentially improve the accuracy of the results. After improving the model’s accuracy, this sensor can be installed on the vehicles in the agricultural field to measure the soil moisture content.

## Figures and Tables

**Table 1:** Comparison of soil moisture values.

Soil Moisture Measured Using Vernier Sensor (VWC)	Soil Moisture Determined from the Microwave Experiment (VWC)
−1.5%	3.14%
−1.5%	3.03%
−1.5%	3.10%
−1.5%	3.13%
2.2%	4.44%
2.2%	5.31%
2.2%	5.45%
19%	26.38%
19%	22.87%
19%	22.89%
25%	28.53%
25%	31.47%
25%	32.87%
37%	40.69%
37%	42.40%
37%	42.68%

**Table 2:** Comparison of soil moisture values for the beach soil from ECP.

Soil Moisture Measured Using Vernier Sensor (VWC)	Soil Moisture Determined from the Microwave Experiment (VWC)
2.6%	−0.422%
2.6%	−1.226%
2.6%	1.783%
26.5%	14.547%
28.0%	15.187%
31.9%	16.45%
38.5%	27.394%

## Data Availability

We collected the data using our own sensor.
